# Lingunite-a high-pressure plagioclase polymorph at mineral interfaces in doleritic rock of the Lockne impact structure (Sweden)

**DOI:** 10.1038/srep25991

**Published:** 2016-05-18

**Authors:** Amar Agarwal, Boris Reznik, Agnes Kontny, Stefan Heissler, Frank Schilling

**Affiliations:** 1 Division of Structural Geology and Tectonophysics, Institute of Applied Geosciences, Karlsruhe Institute of Technology, 76131 Karlsruhe, Germany; 2 Department of Earth Science, Indian Institute of Technology, Roorkee, India; 3 Laboratory of Paleomagnetism, Institute of Geophysics, National Autonomous University of Mexico, 4510 Mexico DF, Mexico; 4 Institute of Functional Interfaces, Karlsruhe Institute of Technology, 76344 Eggenstein-Leopoldshafen, Germany; 5 Division of Technical Petrophysics, Institute of Applied Geosciences, Karlsruhe Institute of Technology, 76131 Karlsruhe, Germany

## Abstract

Lingunite nanocrystals and amorphous plagioclase (maskelynite) are identified at the contacts between augite and labradorite wedge-shaped interfaces in the doleritic rocks of the Lockne impact structure in Sweden. The occurrence of lingunite suggests that the local pressure was above 19 GPa and the local temperature overwhelmed 1000 °C. These values are up to 10 times higher than previous values estimated numerically for bulk pressure and temperature. High shock-induced temperatures are manifested by maskelynite injections into microfractures in augite located next to the wedges. We discuss a possible model of shock heterogeneity at mineral interfaces, which may lead to longer duration of the same shock pressure and a concentration of high temperature thus triggering the kinetics of labradorite transformation into lingunite and maskelynite.

Shock localization is an important issue in many geoscientific fields. *In situ* shock localization can be determined, on a large scale by seismic investigations^1^,^2^,^3^, and at the micrometer scale by using petrological imprints in minerals and rocks^4^,^5^,^6^. For instance, extreme shock deformations conserved in meteorite impacts can be uncovered through identification of high-pressure polymorph transformations and microstructures such as, the shock-induced graphite-diamond phase transition in gneiss from the Ries Crater, Germany^6^. Theoretical studies suggest that the propagation of shock waves across heterogeneous interfaces, such as the interface of different minerals with impedance contrast, may lead to longer duration of the same pressure and higher magnitude of temperature, thus, promoting the kinetics of nucleation of the high-pressure polymorph^6^,^7^,^8^. The high pressure-temperature (P-T) phase transitions in plagioclase are especially sensitive to monitor shock-induced phase transitions in stony meteorites, which resemble in their composition to terrestrial mafic rocks. In particular, lingunite, a high P-T polymorphs of sodic plagioclase has been observed in stony meteorites^9^,^10^,^11^,^12^,^13^ or synthesized in anvil cells by compressing sub-millimeter sized material^14^,^15^,^16^. Transformation of plagioclase into lingunite is accompanied by amorphization of the plagioclase^17^. It is noteworthy that, even though there are 188 confirmed impact structures on Earth^18^, many with feldspar bearing target rocks, there is only one short report on silicate-hollandite in a target rock of the Manicougan impact structure^19^. To our knowledge, transformation of plagioclase into high pressure polymorphs, due to reverberations of shock-wave at heterogeneous mineral interfaces has not been reported so far.

Here we report lingunite and amorphous labradorite (maskelynite) formed probably due to concentration of shock induced T in shocked doleritic basement rocks at the Lockne impact structure, Sweden. This is one of the rare reports on high P-T phase transitions of plagioclase into lingunite at heterogeneous mineral interfaces in impactites.

## Geological setting and sample description

**Figure 1:**
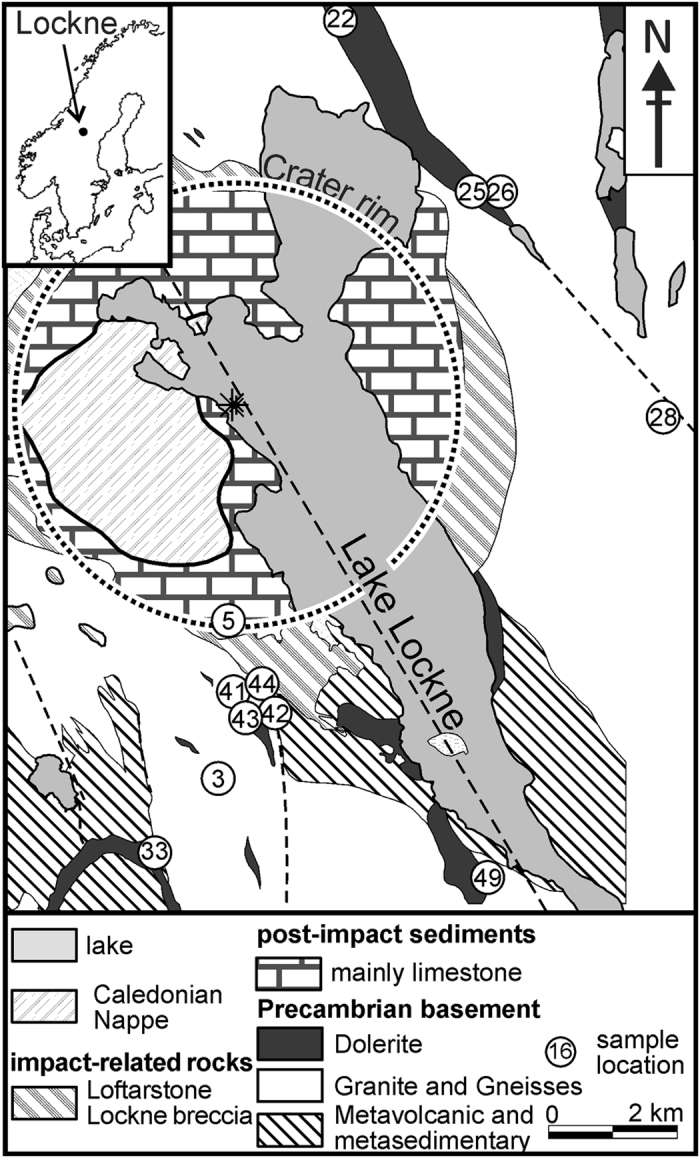
Geological map of the Lockne impact structure showing major lithologies modified after Sturkell and Ormö^60^. The asterisk marks the crater centre. The map is prepared in Corel Draw X6 software.

The Lockne impact structure (c. 14°40′E, 63°00′N) is a well-known impact structure with c. 7.5 km wide inner crater (Fig. 1). The Ordovician (455 Ma) impact occurred in a marine environment with Proterozoic metavolcanic, granitic and doleritic basement rocks overlain by Cambro-Ordovician shale and limestone as target rocks^20^. Numerical modelling indicated formation of the Lockne crater in a 500–700 m deep sea due to a c. 600 m wide projectile, travelling at 15 km/s from the east at 45° from the horizontal^21^,^22^,^23^. Numerical estimates reveal that in 4 to 10 km from the crater centre, the bulk shock pressure and temperature (P-T) range between 0.1 to 3 GPa and 0 to 127 °C^21^. Chromite grains discovered in middle Ordovician limestone in south Sweden were interpreted to represent relics of a fossil L-chondritic meteorite related to a major parent body breakup in the asteroid belt at Ordovician time^24^,^25^. Fragments of this global event enhanced the meteorite flux on Earth for several orders of magnitude in the late solar system history^24^,^25^.

## Results

### Evidences of shock-induced deformation

**Figure 2:**
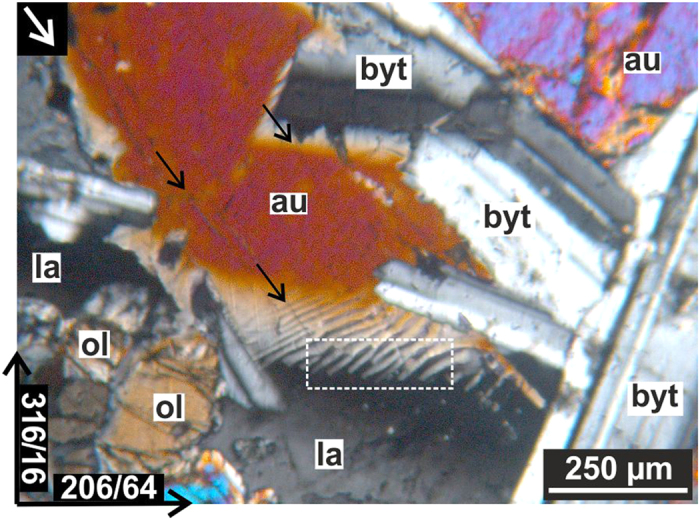
Representative light photomicrograph (cross polarized) of dolerite (sample 49 shown in Fig. 1) composed of augite (au), labradorite (la), olivine (ol) and bytownite (byt) minerals as well as of peculiar alternating augite-labradorite wedges (marked by white rectangle). Note that the shock wave, propagating outward from the crater center (white arrow) and microfractures (black arrows) are parallel with each other. Black arrows with numbers, at the bottom left corner, demonstrate the orientation of the photomicrograph margins.

The dolerites at Lockne impact structure consist of olivine, augite, labradorite and bytownite (Fig. 2). A peculiar microstructure composed of several alternating augite and labradorite wedges (white frame in Fig. 2) is abundant (11 out of 14 dolerite samples; Fig. 1). Such complex, comb like structures have neither been observed at grain boundaries of the other silicates (e.g. between olivine and plagioclase), nor in unshocked rocks from more distant doleritic rocks in the surrounding of the Lockne impact structure e.g.,^26^.

**Figure 3:**
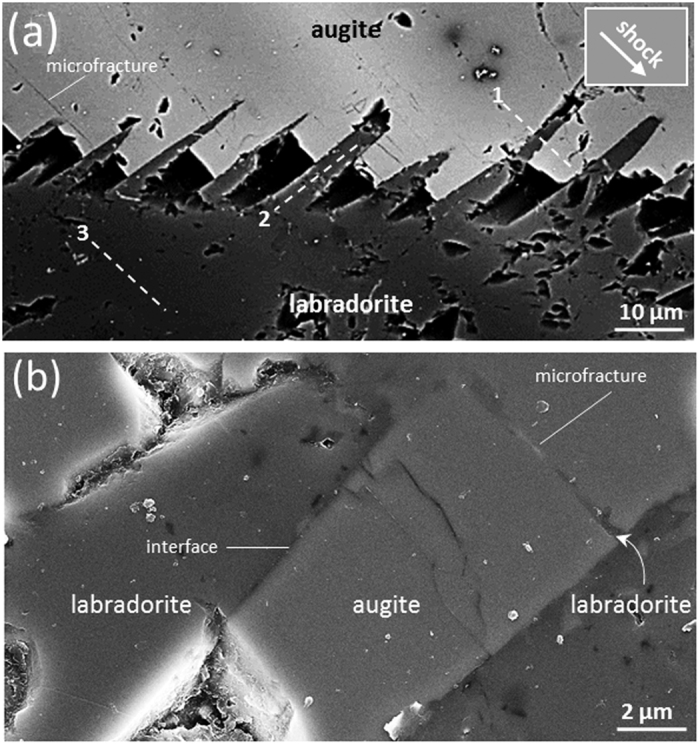
High resolution SEM image of augite-labradorite wedge interfaces. (**a**) Backscattered electron image showing impact generated microfractures oriented parallel with the shock wave propagation direction (white arrow). Dashed lines schematically mark the locations of focused ion beam (FIB) sections for transmission electron microscopy (1) across augite - labradorite wedges interfaces, (2) within labradorite wedge and (3) in bulk labradorite. (**b**) High resolution SEM image of an augite-labradorite wedge interface. Curved arrow points to a microfracture filled with labradorite from the adjacent lamella.

Figure 3 presents detailed SEM observations of the area of the white rectangle in Fig. 2. Boundaries of both labradorite and augite wedges are slightly curved near the labradorite grain (Fig. 3a). Microfractures in augite wedges are at high angles to the wedge shaped structures and are filled with labradorite from adjacent wedges (s. the curved arrow Fig. 3b). This feature is similar to injected labradoritic melt into shock induced fractures in pyroxene described from shocked Shergottites^27^. Microfractures, which are interpreted to be shock-induced, occur in augite as well as in olivine grains (Fig. 2) cf.^61^.

Augite and labradorite grains and their corresponding wedges have comparable chemical composition (Supplementary Table S1). The chemical composition at the labradorite wedge interfaces represent a mixture of augite and plagioclase (Supplementary Table S1). This is due to beam overlap, and because electron exited volume is larger than the electron beam size in SEM-EDX analysis. Therefore we applied additional Raman and high-resolution transmission electron microscopy (HRTEM) studies to investigate the structural and crystallographic changes at the wedge margins.

### Evidences of high-pressure transformation in plagioclase

**Figure 4:**
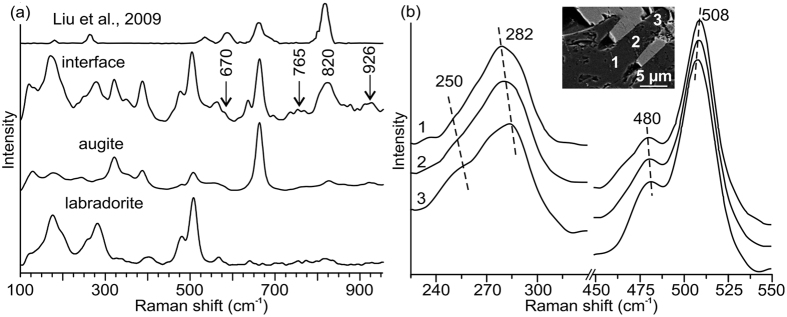
(**a**) Raman spectra from an augite-labradorite wedge interface, and augite and labradorite bulk minerals. Top: Raman spectrum of K-lingunite at *in situ* pressure of 19 GPa from Liu *et al.*^31^. Note that beside the labradorite and augite bands, the spectrum from the interface contains additional bands, at 670, 765, 820 and 926 cm^−1^. The 670, and 820 cm^−1^ bands are comparable to the spectrum of lingunite reported by Liu *et al.*^31^ (**b**) Raman spectra acquired along labradorite wedge. Shifts (dashed lines) and broadening of 262, 282, 480 and 508 cm^−1^ bands increase towards the wedges tip indicating increase in deformation intensity (see also Supplementary Table S2). Inset: SEM image demonstrating area for a spatial resolved Raman scan in the wedge.

Raman spectra from augite, labradorite and their wedge interface were acquired for evidence of phase and/or structural transformation (Fig. 4a). The spectra from bulk labradorite and augite minerals are comparable to that of labradorite and augite reported elsewhere cf.^28^,^29^,^30^ and are in good agreement with our petrographical results (Fig. 2). The spectrum from the augite-labradorite wedge interface shows active bands corresponding to both labradorite and augite (Fig. 4a)^28^,^29^,^30^. This is because the wedges are dipping with respect to the tilted thin-sections and the laser beam excited both the labradorite as well as augite below the surface. Beside these bands, the spectrum from the interface shows additional bands at 670, 765, 820 and 926 cm^−1^. The intense 820 cm^−1^ and weak 670 cm^−1^ bands are neither characteristic of augite nor labradorite cf.^28^,^29^,^30^. However, these bands compare well with the *K*-lingunite spectrum reported^31^ by Liu *et al.*^31^ (Fig. 4a; Supplementary Figure S2). The 820 cm^−1^ band is most intense in the lingunite spectrum and varies between 760 and 900 cm^−1^. The band, characteristic of SiO_6_ octahedral stretching vibrations, corresponds to splitting of A_1g_ mode^31^. The broad shape of the 820 cm^−1^ band may be attributed to either a deformed crystal lattice and/or semi-amorphous material with many, variably oriented nanocrystals^32^. Such changes may also contribute to the weak and broad 765 cm^−1^ band, as it is the strongest band of the undeformed lingunite^31^ and compares closely, within few wave numbers, to that of KAlSi_3_O_8_-hollandite (*K*-lingunite) observed in Sixiangkou L6 chondrite^9^. The broad 926 cm^−1^ is similar to that of either diaplectic glass^33^ or maskelynite^34^. However, the spectrum of diaplectic glass and maskelynite are very similar^33^,^34^. Thus, Raman spectrum alone cannot discriminate between diaplectic glass and maskelynite and additional HRTEM analysis is employed. Further important evidence for impact-induced deformation in the labradorite wedge is manifested by the evolution of 282 cm^−1^, 480 cm^−1^ and 508 cm^−1^ bands (Fig. 4b). These bands are assigned to the external lattice modes, a mixed Si-O-Si (or Si-O-Al) bending/stretching, A_g_ vibrational mode respectively^28^, is studied as a function of distance from the bulk labradorite grain (Fig. 4b). The fact that the three active bands are absent in the typical Raman spectrum of shock-densified anorthite glass (cf. Reynard *et al.*^34^) indicates that, the wedges may be composed of crystalline labradorite and amorphous labradorite may be present only along the wedge interface. Firstly, the bands show a gradual and systematic shift either to a higher or a lower value on moving towards the wedge tip (Fig. 4b), which may be attributed to anisotropic straining, common in triclinic minerals such as labradorite^35^. Secondly, the bands broaden towards the wedge tip (Supplementary Table S2); for example the full width at half maxima (FWHM) of the bands near the wedge tip (pt. 3 in inset of Fig. 4b) is 12 to 15% higher than that near the bulk labradorite grain (pt. 1 in inset of Fig. 4b). Similar to the shifts in active bands, the shoulder at 250 cm^−1^ also demonstrates shifts to lower value (Fig. 4b). The gradual broadening and shift may imply that the labradorite in the wedge is deformed and the deformation increases towards the wedge tip. In this context, it is important to note that Liu *et al.*^31^ reported deformed crystal lattice showing similar Raman spectrum (Supplementary Figure S2) from static experiments. Obviously, after loading and release in the static experiments, the crystal lattice gets sufficient time to relax back elastically to its original state. In contrast, the high-rate meteorite shock impact provokes rapid plastic deformation^6^,^7^. The crystal, thus, retains some residual strain, which is represented in the present Raman results, for example the position and broad shape of the 820^−1^ cm band (Figs 2 and 4a). To validate these results HRTEM investigations were done.

### Nanoscale deformation at augite-labradorite wedges contact zone

Further structural investigations were done at the nm-scale using HRTEM. Such investigations may answer the question “What type of shock induced lattice defects, if present, accompany the deformation in lingunite and labradorite?” For this purpose focused ion beam (FIB) sections prepared from bulk labradorite, as shown in Fig. 3a, are compared with those prepared from the labradorite wedge and the labradorite-augite wedge contact zone.

**Figure 5:**
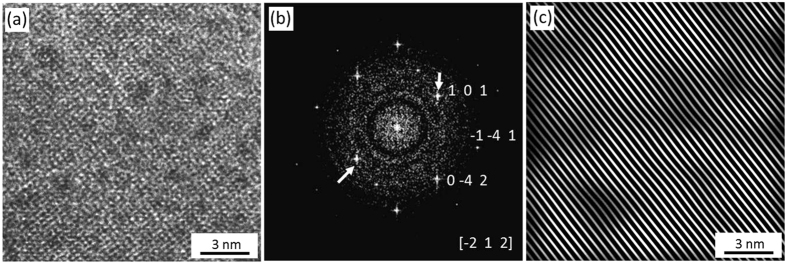
HRTEM analysis of the bulk labradorite. (**a**) Regularly ordered atoms forming perfect crystal lattices. (**b**) FFT pattern of (**a**) demonstrating sharp spots and [−212] labradorite axis. (**c**) IFFT pattern generated using 101 frequencies shown by arrows in (**b**). Note the perfectly aligned augite lattice planes.

The HRTEM image of the bulk labradorite displays groups of individual atoms (Fig. 5a). The corresponding Fast Fourier Transformation (FFT) pattern (Fig. 5b) contains sharp spots whose reciprocal allocation is in good accordance with the interplanar distances reported for labradorite^36^. Zone axis of the FFT pattern, i.e., the axis along the incident electron beam, compares closely with the [−212] labradorite axis. The Inverse Fast Fourier Transformation (IFFT) pattern of {101} lattice planes of labradorite, which are also the active deformation (sliding) planes^37^, demonstrates (101) lattice fringes without any amorphous polymorph in the bulk labradorite (Fig. 5c). Absence of amorphous polymorph implies that the FIB preparation did not produce any artefacts such as material amorphization.

**Figure 6:**
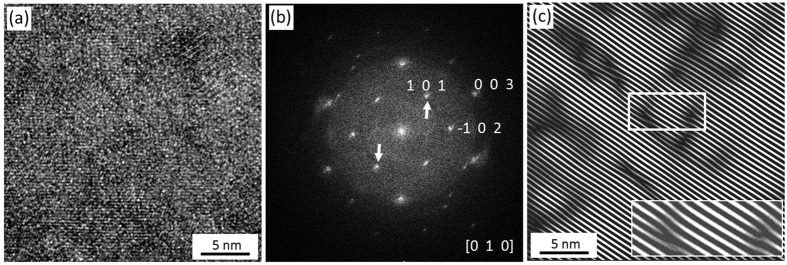
HRTEM analysis of the labradorite wedge. (**a**) Labradorite with overlapping and slightly rotated crystalline domains. (**b**) FFT pattern of (**a**) showing distorted spots in a diffuse background. (**c**) IFFT pattern obtained by using of 101 frequencies shown by arrows in (**b**). Numerous edge dislocations in (101) planes are observed, two of which are enlarged in the inset.

In contrast to the well-resolved atomic domains in bulk labradorite (Fig. 5), the labradorite wedge demonstrates superposed and overlapping domains (Fig. 6). The [010] zone axis is normal to the TEM section, i.e., along the incident electron beam (Fig. 6). The corresponding FFT pattern is composed of elongated diffraction spots and a diffuse background (Fig. 6b). Firstly, the streaking shape of diffraction spots is quite common due the double diffraction^38^,^39^. For example, in the case of very perfect single crystals or overlapping regular arrays of precipitates the diffraction symmetry conditions may be broken. As a result, the multiple electron scattering appears in from of forbidden satellites or double diffracted spots located outside the main Laue spots. The presence of closely spaced twins may as well provoke streaking of the diffraction spots^38^,^39^. In this situation, the additional spots should be accompanied by diffuse streaks, which also commonly appear outside the main Laue spots. In the case of the labradorite wedges, the additional spots are very weakly developed (Fig. 6b). Furthermore, the material from the labradorite wedges is not a perfect single crystal but characterized by deformation features in form of shifted bands (Fig. 4) and overlapped nano-domains (Fig. 6a). Therefore, double diffraction may not be the main reason responsible for the spot streaking. However the presence of twinned regions within the labradorite wedges cannot be completely ruled out. Finally, the steaking of diffraction spots or the so called Laue asterism is commonly associated with the occurrence of continuous misorientations in the strained crystals subdivided into sets of subgrains^38^. The asterism were abundantly observed in a broad range of materials including deformed metals^38^,^39^,^40^ and impactites^41^,^42^,^43^,^44^. For instance, using micro X-ray diffraction (μXRD), Flemming^44^ observed similar diffraction asterism in strained quartz grains from La Malbaie quartzite, Quebec and in shocked clinopyroxenes from Shergottite meteorite, NWA 3171. It appears reasonable to conclude that in case of the labradorite wedge (Fig. 6b), the distortion of the diffraction spots may be attributed to the deformation-related asterism. This implies certainly that the labradorite in the wedges is much more deformed by shock metamorphism than the bulk labradorite (cf. Fig. 5b). This statement is consistent with their electron optical appearance displaying mosaic domains (Fig. 6a). Furthermore, contrary to the undeformed bulk labradorite (Fig. 5c), the IFFT pattern of labradorite in the wedges shows numerous edge dislocations within (101) planes (Fig. 6c) and confirms its deformed state.

**Figure 7:**
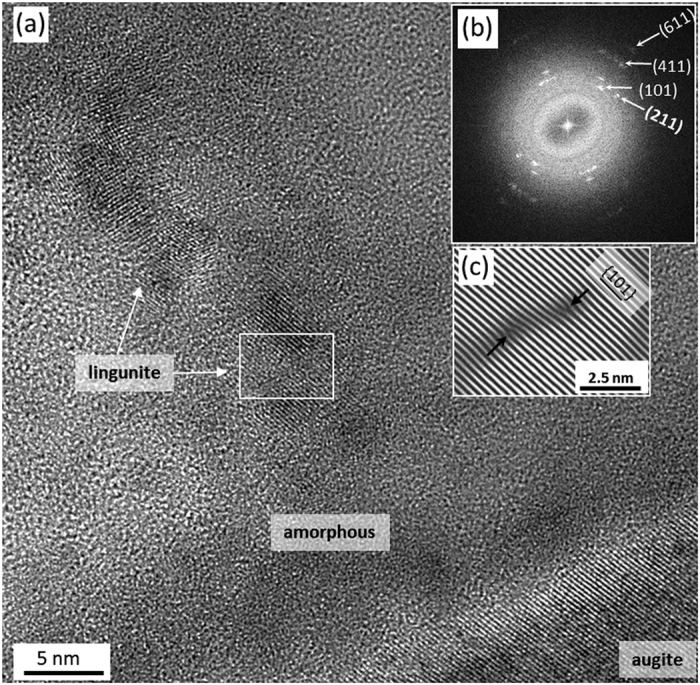
HRTEM analysis of the augite-labradorite wedge interface. (**a**) Well-crystallized augite (right-down) and amorphous matrix containing lingunite nanocrystals. (**b**) FFT pattern from a lingunite area. Numbers indicate indices of Bragg reflection planes. (**c**) IFFT pattern of the area in the white frame displaying two edge dislocations (s. black arrows) in {101} planes.

At the labradorite - augite wedge interface, the well-ordered augite crystal is bordering amorphous matrix (Fig. 7a). Randomly oriented, 60 to 150 nm^2^ in size, nanocrystals are embedded in the amorphous matrix (Fig. 7a). These nano-crystals are rounded with serrated grain boundaries, suggesting partial melting after crystallization. Augite demonstrates perfectly aligned planes, 2.98(3) Å from each other. This distance is in accordance with values published for (−221) planes of augite^36^. Perfectly crystallized augite planes imply that the FIB preparation did not produce artefacts. We, therefore, used augite as an inner reference material to investigate various phases of the nanocrystals. Figure 7b is a FFT pattern from the area containing nanocrystals embedded in the amorphous matrix. Here the diffuse Debye-Scherrer rings are associated with a diffuse halo that indicates a crystalline and an amorphous (glass-like) material. Using augite interplanar distance of 2.98(3) Å as a reference, we obtained Bragg diffraction rings corresponding to the interplanar distances of 2.62(3), 2.26(3), 1.73(3) and 1.33(4) Å. These distances are comparable to the interplanar distances between (101), (211), (411) and (611) planes of lingunite^10^,^13^,^15^,^16^. The occurrence of lingunite in the contact zone is also confirmed by a selected area electron diffraction pattern (Supplementary Figure S5, Table S3). Unlike the perfectly aligned (−221) planes of augite, (101) planes of lingunite show numerous edge dislocations (cf. Fig. 7a,c). Similar dislocations in (101) planes are common in the labradorite wedge (Fig. 6c).

The HRTEM results, revealing more intensely deformed labradorite in the wedge as compared to the bulk (Figs 5, 6, 7) are in a good agreement with our Raman results (Fig. 4a). Randomly oriented and deformed lingunite nanocrystals embedded in an amorphous matrix (Fig. 7a) explain the broadening and shift of Raman bands (Fig. 4b). It is significant to note that the lingunite nanocrystals and amorphous labradorite are detected only in a zone along the augite-labradorite wedges contact and not in the middle of the wedge or in the bulk labradorite grain (Figs 5 and 6). Origin of the lingunite nanocrystals is, therefore, related and restricted to processes active at the augite-labradorite interface.

## Discussion

The dolerites from the basement of the Lockne Impact structure are part of the Central Scandinavian Dolerite Group^45^. Previous workers have studied this dolerite in extensive detail^46^,^47^,^48^,^49^,^50^, and few reports have suggested that the dolerite experienced thrust related very low grade metamorphism^26^,^51^. Greiling *et al.*^26^ studied the same, but unshocked, Mesoproterozoic dolerite dykes from Orkulla, Sweden, about 200 km north from the Locke impact structure. However, none of these studies report the pyroxene-plagioclase wedges reported in present study. Therefore, it is very unlikely that the augite-plagioclase wedges were a result of magmatism or metamorphism. Instead, we suggest that they formed due to passing of the shock-waves. Occurrence of labradorite in microfractures within augite wedges (Fig. 3b) strongly suggests that the shock-wave induced injection of molten labradorite into the fractures of augite. Previous studies on shocked L-chondrite and SNC meteorite^52^ and Shergottites^27^ elucidate that such microfractures develop during the decompression. Since the labradorite wedges are barren of microfractures (Fig. 3), it is reasonable to presume that the labradorite melt injection solidified during the decompression stage.

This idea of shock-induced transformations is strongly supported by the observation of high-pressure plagioclase phase lingunite and labradorite glass (Figs 6 and 7). Transformation of plagioclase (labradorite) at the interface of minerals with contrasting mechanical properties may be explained by the processes leading to longer duration of the high pressure regime and shear induced melting^5^,^6^,^7^,^8^,^52^. Theoretical studies suggest that the propagation of shock waves across heterogeneous interfaces, such as the contact zone of minerals with impedance contrast, may lead to longer duration of the same pressure, and higher magnitude of temperature thus promoting the kinetics of nucleation of the high-pressure polymorph^7^,^8^. In the present case the augite-labradorite interface representing a heterogeneity, due to a large contrast in mechanical strength, ~15%, between augite and labradorite (Poisson’s ratio are 0.293 and 0.243, respectively), facilitate the formation of lingunite and labradorite glass at a very small scale (Figs 6 and 7).

It is a well-known fact that the duration of shock-wave is much longer in natural impact than in experiments^7^. Impedance contrast leading to shock wave heterogeneity assisted in melting of labradorite. We predict that during the shock-wave imparted compression, labradorite melt was injected in the pre-existing augite fractures forming alternating augite-labradorite wedges. Sharp and de Carli^7^ predict a similar situation where, due to shock heterogeneity former open cracks would become larger melt veins. Moreover, similar injections of melt veins are also reported from Shergottites^27^. The labradorite melt, injected in the pre-existing augite fractures, solidified into glass and lingunite nano-crystals during the decompression. The labradorite glass has a Raman spectrum similar to both maskelynite and diaplectic glass. However, the labradorite glass lacks features such as cleavage, cracks and shock induced fractures, which are characteristic of diaplectic glass^52^. Similar to the offshoots of maskelynite into shock-induced fractures of neighbouring olivine in Shergottites^52^, shock-induced fracture wedges in augite are filled with labradorite glass (Fig. 3b). Such offshoots are highly uncharacteristic of diaplectic glass. We may thus conclude that the glass in the labradorite wedges and shock-induced fracture of augite is maskelynite. Maskelynite was first defined by Tschermak^53^,^54^. As suggested by Chen and El Goresy^52^, and El Goresy *et al.*^27^, maskelynite is not diaplectic plagioclase glass formed by solid-state transformation, but a dense quenched glass. We suggest that, shock-impedance at mineral interfaces, with stark difference in densities, may induce reverbrations of the shock-waves at the interfaces, producing high temperatures at same high pressure magnitude leading to melting of plagioclase. This was followed by injection of the dense liquid in shock-induced fractures in neighbouring augite followed by quenching under high pressure.

The presence of nanometer-sized lingunite islands in the labradorite glass strongly suggest longer duration in the decompression stage thus allowing lingunite nuclei to crystallize. Kubo *et al.*^17^ discuss that the breakdown/transformation of plagioclase into either a high P-T crystalline or amorphous polymorph depends upon the kinetics in such a way that, when the transformation into high P-T crystalline polymorph is sluggish, plagioclase transforms into amorphous matter. A similar temperature difference is predicted between melt vein and host rock of the Tenham L6 chondrite^55^. Here, extremely high temperatures in the veins triggered the formation of lingunite nanocrystals embedded in an amorphous matrix^55^. It has been shown that KAlSi_3_O_8_ and NaAlSi_3_O_8_ transform at 1026 °C into lingunite at 12 GPa and 21 to 24 GPa, respectively^9^,^14^,^15^. Previous numerical estimates predict that, in the studied area, 4 to 10 kms from the crater centre, the shock P-T range between 0.1 to 3 GPa and 0 to 127 °C^21^. Sharp and de Carli^7^ suggest that, the initial peak pressure, and therefore the shock temperature, in the shock front can vary by as much as an order of magnitude from grain to grain or even within a single mineral grain, depending on details of the local environment. Generally theoretical dynamic estimates and numerical modelling never predicted or discussed the possibility of selective shock-induced melting, fractional crystallization of dense monomineralic phases and quenching of high-pressure liquid products^27^,^35^,^56^,^57^,^58^. The estimates of Lindstrom *et al.*^21^ are, thus, very rough and do not account for microscale fluctuations in peak shock P-T.

In summary, high-pressure plagioclase polymorphs along interfaces between augite and plagioclase wedges have been observed in dolerite of the Lockne impact structure. No evidence was found in this study for a chemical exchange between the phases at the mineral interfaces. More studies, however, are needed to investigate other heterogeneous mineral associations, which have experienced shock waves. Thus, it must be underlined that a combination of Raman spectroscopy and HRTEM is a powerful tool to study shock-induced phase transitions providing valuable information on pressure–temperature–time history of impact shock events.

## Methods

### Petrology

From each oriented dolerite block sample oriented polished thin sections were prepared and petrological observations were carried out using an optical polarizing Leitz Othoplan microscope.

High-resolution morphological investigation was performed through a scanning electron microscope (SEM), LEO 1530 of Gemini, equipped with an EDX-detector for chemical analysis.

### Raman spectroscopy

In order to reveal possible shock-induced phase transformations, micro-Raman spectrometry was employed. A Bruker SENTERRA Raman spectrometer (Bruker Optics, Ettlingen, Germany) based on an Olympus BX-51 microscope (OLYMPUS Co. Tokyo Japan) provided insights into structures on the micrometre scale. A frequency doubled NdYAG Laser, λ = 532 nm, operated at 5 mW power, served as the excitation source. The beam was focused through an objective, Olympus 100X, NA 0.8, in a 1 μm spot on the sample surface. An integration time of 80 s with 2 co-additions (2*40 s) was used to scan an area about 190*90 μm. The obtained spectra were processed and baseline corrected using Bruker OPUS® software Ver. 7.2.

### High-resolution electron microscopy

Nanostructural investigations on rock thin sections were carried out by a Philips CM 200 FEG/ST TEM operated at 200 kV. The Fast Fourier Transformation (FFT) of HRTEM images were carried out using ImageJ software^59^. TEM sections were prepared using focused iron beam (FIB) milling station FEI Dual Beam Strata 400S with a Ga+ cathode. Here the area of interest was coated with a 3 μm thick conductive platinum layer. Thereafter, sample was cut from the thin-sections by a 30 kV, 6.5 nA Ga ion beam. Later, the sample was thinned by a 30 kV, 26 pA Ga ion beam and two to three, 5 to 50 nm thick, windows were prepared to check FIB induced damage and amorphization. In the final step, the sample was polished/cleaned with a 5 kV (71 pA ion current). The step is essential to remove its crust, which is commonly damaged by the high energy ion beam. We propose that the polishing was competent as the augite, present above lingunite, and therefore closer to the ion beam source is crystalline (Fig. 7).

It is well-known that plagioclases are sensitive to amorphization due to irradiation by electron beam^37^. We avoided the amorphization by using sample holder cooled by liquid nitrogen, and smaller exposure time of about 10–20 ms. A condenser aperture of 100 μm at a spot size of ≥300 nm was used for initial selective area electron diffraction (SAED) image and general TEM investigations. Usually much higher energy is transmitted to the sample during high resolution TEM (HRTEM) investigations. Therefore, for HRTEM the beam was first focused at a location away from the area of interest and only then the investigations were carried out.

## Additional Information

**How to cite this article**: Agarwal, A. *et al.* Lingunite-a high-pressure plagioclase polymorph at mineral interfaces in doleritic rock of the Lockne impact structure (Sweden). *Sci. Rep.* **6**, 25991; doi: 10.1038/srep25991 (2016).

## Supplementary Material

Supplementary Information
