# Supplementary material for: Lingunite-a high-pressure plagioclase polymorph at mineral interfaces in doleritic rock of the Lockne impact structure (Sweden)
Source: Sci Rep. 2016 May 18;6:25991. doi: 10.1038/srep25991 (PMC4870623; doi:10.1038/srep25991)
Supplement: Supplementary Information [file srep25991-s1.doc]

**Supplementary Materials for**

**Lingunite-a high-pressure plagioclase polymorph at mineral interfaces in doleritic rock of the Lockne impact structure (Sweden).**

Amar Agarwal^1,2^, Boris Reznik^1*^, Agnes Kontny^1^, Stefan Heissler^3^, Frank Schilling^4^

^1^ Division of Structural Geology and Tectonophysics, Institute of Applied Geosciences, Karlsruhe Institute of Technology, 76131 Karlsruhe, Germany.

^2^ Department of Earth Science, Indian Institute of Technology, Roorkee, India.

^3^ Institute of Functional Interfaces, Karlsruhe Institute of Technology, 76131 Karlsruhe, Germany.

^4^ Division of Technical Petrophysics, Institute of Applied Geosciences, Karlsruhe Institute of Technology, 76131 Karlsruhe, Germany.

*Corresponding author: [boris.reznik@kit.edu](mailto:boris.reznik@kit.edu)

**This file includes:**

Supplementary Text

Supplementary Figures S1 to S5 and Supplementary Table S1 to S3

EDX analysis

**Supplementary Table S1.** Comparison of weight percentage of major oxides between labradorite (1), augite (3), the respective lamellae (2, 4) and the labradorite-augite lamellae contact zone (5). The results are from the EDX study during SEM. Schematic positions of EDX analyses are shown in Supplementary Figure S1.

| **Oxide** | Labradorite (la) | | | | Augite (au) | | | | au-la lamellae interface | |
| --- | --- | --- | --- | --- | --- | --- | --- | --- | --- | --- |
|  | Position 1 | | Position 2 | | Position 3 | | Position 4 | | Position 5 | |
|  | Weight % | error weight% | Weight % | error weight% | Weight % | error weight% | Weight % | error weight% | Weight % | error weight% |
| **Na_2_O** | 5.14 | +/- 0.12 | 4.62 | +/- 0.07 | --- | --- | --- | --- | 5.22 | +/- 0.14 |
| **MgO** | --- | --- | --- | --- | 11.37 | +/- 0.16 | 11.76 | +/- 0.13 | 1.27 | +/- 0.09 |
| **Al_2_O_3_** | 25.71 | +/- 0.19 | 25.33 | +/- 0.10 | 2.43 | +/- 0.08 | 2.3 | +/- 0.07 | 20.25 | +/- 0.13 |
| **SiO_2_** | 59.1 | +/- 0.28 | 60.13 | +/- 0.15 | 50.63 | +/- 0.18 | 50.9 | +/- 0.16 | 61.73 | +/- 0.22 |
| **K_2_O** | 0.8 | +/- 0.07 | 0.81 | +/- 0.04 | --- | --- | --- | --- | 0.49 | +/- 0.08 |
| **CaO** | 9.24 | +/- 0.24 | 9.12 | +/- 0.07 | 23.33 | +/- 0.21 | 21.5 | +/- 0.27 | 9.28 | +/- 0.27 |
| **Fe_2_O_3_** | --- | --- | --- | --- | 11.07 | +/- 0.61 | 12.28 | +/- 0.56 | 1.76 | +/- 0.26 |
| **TiO_2_** | --- | --- | --- | --- | 1.16 | +/- 0.11 | 1.26 | +/- 0.11 | --- | --- |


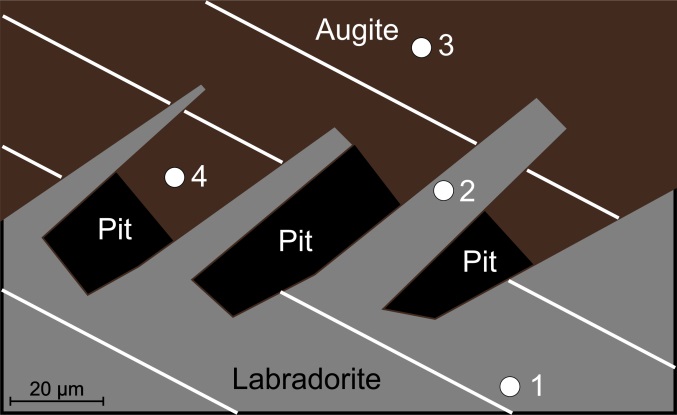


**Supplementary Figure S1.** Schematic representation of alternating augite and labradorite lamellae with impact generated microfractures (white lines) (compare with figure 3a), showing position of EDX measurements.

Raman spectroscopy

**
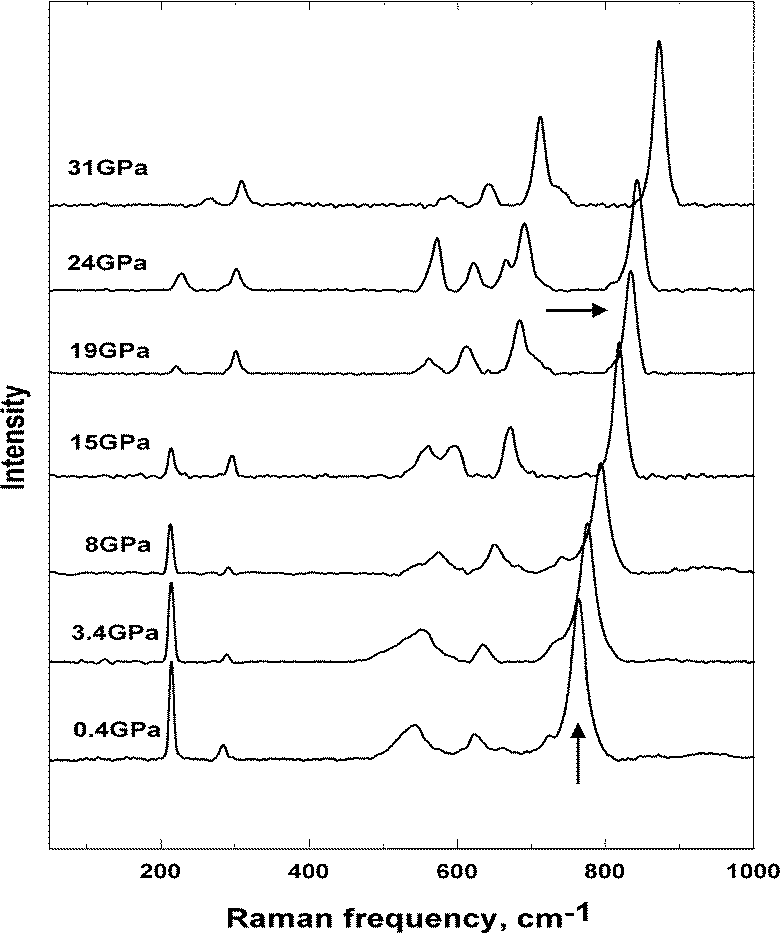
**

**Supplementary Figure S2.** Raman spectra of K-lingunite as a function of pressure at room temperature from Liu et al.^1^. Note the shift of the band labeled by the arrow.

It is important to mention that the spectrum from the contact zone is compared with that of K-lingunite, instead of lingunite (Figure 4a). This is because the lingunite spectrum at high P-T is not available, moreover the lingunite and K-lingunite have similar Raman spectrum ^2^. Due to the plastic deformation of the lingunite nano-crystals and the resulting shift of Raman bands, the spectrum from the contact zone is more similar to that at high P-T rather than ambient P-T.

**Supplementary Table S2.** Evolution of Raman bands with respect to position in the labradorite lamella. Note the increase in Full Width at Half Maxima (FWHM) and shift in position on moving towards lamellae tip. See Figure. 4b inset for position of analysis points 1, 2 and 3.

| Position 1 | | Position 2 | | Position 3 | |
| --- | --- | --- | --- | --- | --- |
| peak (cm^-1^) | FWHM | peak (cm^-1^) | FWHM | peak (cm^-1^) | FWHM |
| 281 | 36.536 | 285 | 38.133 | 288 | 42.072 |
| 466 | 9.977 | 465 | 10.625 | 463 | 11.497 |
| 498 | 17.486 | 498 | 17.790 | 497 | 19.587 |

Calculating the direction of shock wave with respect to the photomicrograph


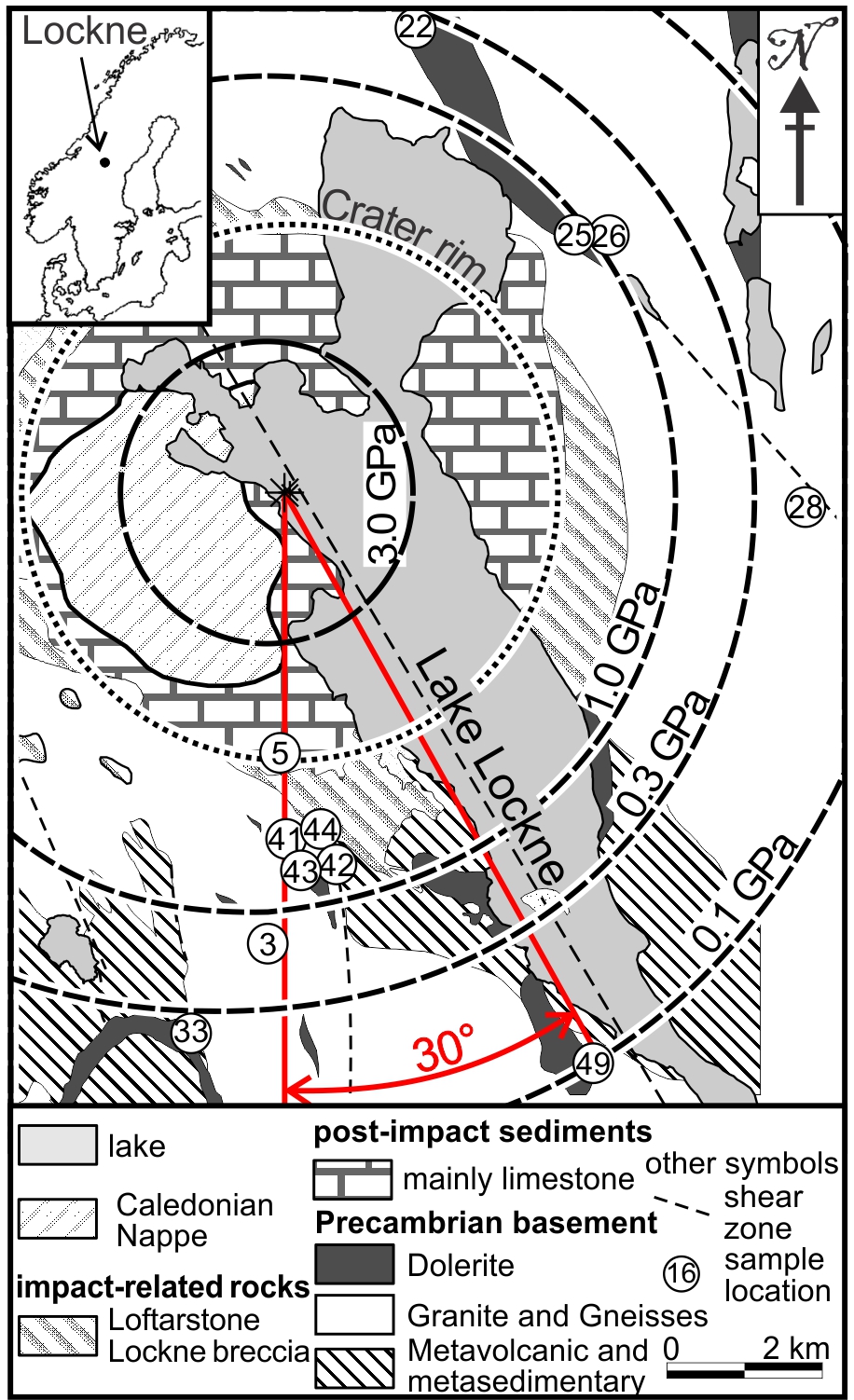


**Supplementary Figure S3.** Geological map of the Lockne impact structure showing that the sample 49 is located S30°E from the center of the impact crater. The map is prepared in Corel Draw X6 software.


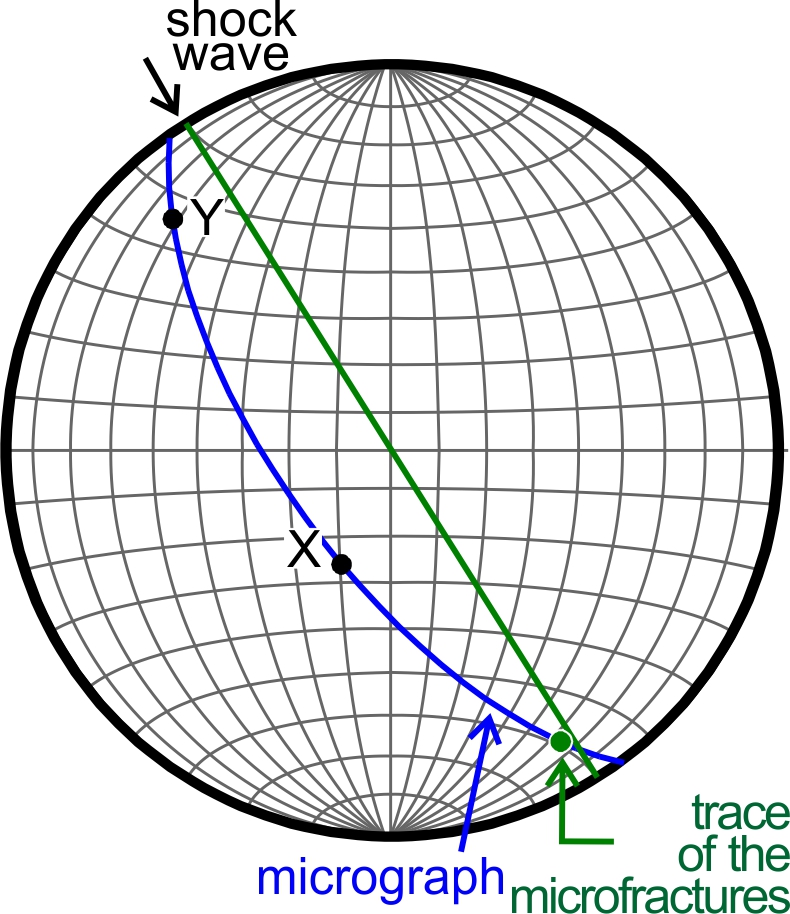


**Supplementary Figure S4.** Stereonet demonstrating the orientation of the above micrograph (blue arc), which was determined using ‘X’ and ‘Y’. The stereonet also shows the direction of shock wave propagation (black arrow), which is towards S30°E with respect to the photomicrograph. The rake of ‘the trace of microfracture’ was calculated with respect to ‘X’ in the above micrograph and is shown with green point.

Transmission electron microscopy (TEM) coupled with selected area electron diffraction (SAED)


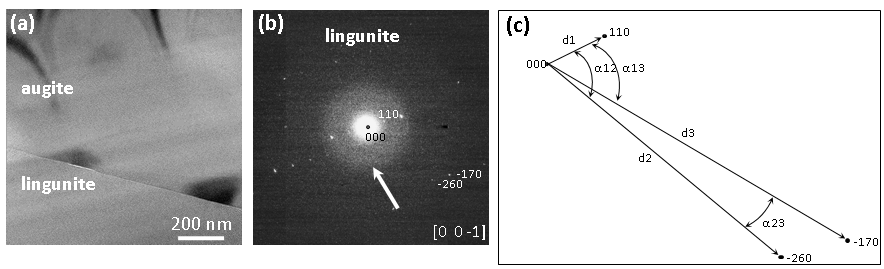


**Supplementary Figure S5.** (a) TEM image of augite-labradorite lamella contact zone. (b) SAED pattern from the labradorite area composed of diffuse white hallo (marked by the arrow) representing diffraction from amorphous phase as well as sharp diffraction spots originating from overlapping nanocrystals. (c) Schematics representing characteristic diffraction spots from a single crystal. After calculating the interplanar distances d and angels α the spots are assigned as diffraction from [00-1] of lingunite, which is parallel to the electron beam (also see Supplementary Table S3).

Supplementary Figure S3 illustrates TEM-SAED analysis of an augite-labradorite lamellae interface. To minimize sample damage by e- beam, short exposure time of ~30 ms, was used while acquiring SAED patterns. The SAED pattern generated from labradorite area (Supplementary Figure S3b) contains, a diffuse ring (marked by an arrow), originating from the amorphous phase, and sharp overlapping spots originating from a nanocrystalline phase. In Supplementary Figure S3b, a single diffraction pattern with characteristic diffraction vectors and ‘hkl’ indices is selected, which is schematically represented in Supplementary Figure S3c. Supplementary Figure S3c also shows correlation between the diffraction vectors (d1, d2 and d3), and angles (α13, α23 and α12) between them. Interplanar distances D and angles between diffraction spots were calculated using lattice parameter of tetragonal hollandite, a = 9.873 Å and c = 2.851 Å, reported by Botkovitz, et al. ^3^ (Supplementary Table S3).

**Supplementary Table S3. Analysis of the SAED pattern shown in Supplementary Figure S3b.**

|  | D1 | D2 | D3 | α13 | α23 | α12 | H1 | K1 | L1 | H2 | K2 | L2 | H3 | K3 | L3 | U | V | W |  |
| --- | --- | --- | --- | --- | --- | --- | --- | --- | --- | --- | --- | --- | --- | --- | --- | --- | --- | --- | --- |
| Calc. | 6.55 | 1.46 | 1.31 | 53.1 | 10.3 | 63.4 | 1 | 1 | 0 | -2 | 6 | 0 | -1 | 7 | 0 | 0 | 0 | -1 |  |
| **Obs.** | **6.55** | **1.41** | **1.28** | **53.1** | **8.2** | **61.3** | 1 | 1 | 0 | -2 | 6 | 0 | -1 | 7 | 0 | 0 | 0 | -1 |  |

D ‘(1, 2, 3)’- interpalanar distances, in Å, ‘α (1 3, 2 3, 1 2)’- angles in degrees, HKL – plane indices, UVW- indices of the resulting zone axis. The calculated values were obtained using 82-1450 powder diffraction file of a tetragonal hollandite, a = 9.873 Å and c = 2.851 Å, reported by Botkovitz, et al. *^2^*.

References

1. Liu, L., Lin, C.-C., Yung, Y. J., Mernagh, T. P. & Irifune, T. Raman spectroscopic study of K-lingunite at various pressures and temperatures. *Phys. Chem. Miner.* **36,** 143–149 (2009).

2. Liu, L.-G. & El Goersy, A. High-Pressure Phase Transitions of the Feldspars, and Further Characterization of Lingunite. *Int. Geol. Rev.* **49,** 854–860 (2007).

3. Botkovitz, P., Brec, R., Deniard, P., Tournoux, M. & Burr, G. Electrochemical and Neutron Diffraction Study of a Prelithiated Hollandite-Type Li x MnO 2 Phase. *Mol. Cryst. Liq. Cryst. Sci. Technol. Sect. A. Mol. Cryst. Liq. Cryst.* **244,** 233–238 (1994).
